# Management of severe bimaxillary crowding using unilateral molar extraction and miniscrew-assisted posterior distalization: A case report

**DOI:** 10.1097/MD.0000000000046683

**Published:** 2025-12-12

**Authors:** Viet Anh Nguyen, Thi Quynh Phuong Vo

**Affiliations:** a Faculty of Dentistry, Phenikaa University, Hanoi, Vietnam; b Private Practice, Viet Anh Orthodontic Clinic, Hanoi, Vietnam.

**Keywords:** class III malocclusion, miniscrew-assisted distalization, orthodontic anchorage, orthodontic biomechanics, unilateral molar extraction

## Abstract

**Rationale:**

Unilateral extraction of the compromised maxillary first molar combined with miniscrew-assisted posterior distalization provides a focused, non-surgical approach to correct asymmetrical Class III subdivision and severe bimaxillary crowding while preserving soft-tissue balance.

**Patient concerns:**

A 26-year-old female with severe bimaxillary crowding, unilateral Class III subdivision, mandibular midline deviation, and a pulp-necrosis maxillary left first molar, seeking a minimally invasive solution without surgery or prosthetic rehabilitation.

**Diagnoses:**

A unilateral dental Class III subdivision malocclusion, severe mandibular and moderate maxillary crowding, right mandibular midline deviation, a poor-prognosis necrotic maxillary left first molar, mild lip protrusion with a slightly convex profile, and impaction of all third molars.

**Interventions:**

The treatment plan involved the extraction of the maxillary left first molar, followed by space closure using conventional anchorage. This was combined with miniscrew-assisted distalization of the maxillary right and mandibular left posterior segments to retract the maxillary dentition, correct the dental midline discrepancy, and create space for alleviating bimaxillary crowding. Temporary anchorage devices were employed to facilitate controlled tooth movement and to minimize undesirable side effects such as incisor tipping and anchorage loss.

**Outcomes:**

After 17 months of active treatment, the patient achieved a Class I canine relationship, coincident dental midlines, ideal overjet and overbite, and a notably improved facial profile. The mandibular midline deviation was corrected via unilateral distalization using buccal-shelf miniscrews, while the left posterior maxillary segment was protracted to substitute the extracted molar. From a soft tissue perspective, lip protrusion was reduced, and overall facial balance was enhanced.

**Lessons:**

This case underscores the clinical effectiveness of combining asymmetric molar extraction with skeletal anchorage mechanics as a non-surgical and non-prosthetic treatment alternative for adult patients with asymmetrical Class III malocclusion and compromised molars.

## 1. Introduction

Unilateral extraction strategies, though less commonly employed than bilateral protocols, have gained attention in orthodontics as a conservative and targeted solution for managing dental asymmetries and arch-length discrepancies. Particularly in adult patients with unilateral malocclusions, midline deviation, or compromised molars, the removal of a single posterior tooth offers a viable alternative to full arch extractions or orthognathic surgery.^[1,2]^ When combined with miniscrew-assisted mechanics, unilateral extraction allows for controlled space closure, midline correction, and improved arch coordination without excessive reliance on patient compliance or reciprocal tooth movement.^[3]^

Miniscrew anchorage has further expanded the biomechanical capabilities of unilateral distalization. Temporary anchorage devices (TADs), particularly those placed in buccal shelf or palatal regions, provide reliable skeletal anchorage to facilitate en-masse distalization or segmental control without compromising anterior esthetics or occlusal function.^[4]^ This is especially advantageous in adult cases where skeletal discrepancies are mild and the primary treatment objectives involve resolving crowding, correcting asymmetry, and improving soft tissue balance.

The present case report illustrates the clinical application of unilateral molar extraction combined with miniscrew-supported distalization mechanics in an adult female patient presenting with dental asymmetry, bimaxillary crowding, and a poor-prognosis maxillary first molar. The treatment outcome demonstrates that appropriate case selection combined with meticulous biomechanical planning can lead to successful orthodontic results without the need for prosthetic rehabilitation or additional extractions for space creation.

## 2. Case presentation

### 2.1. Diagnosis and etiology

**Figure 1.**
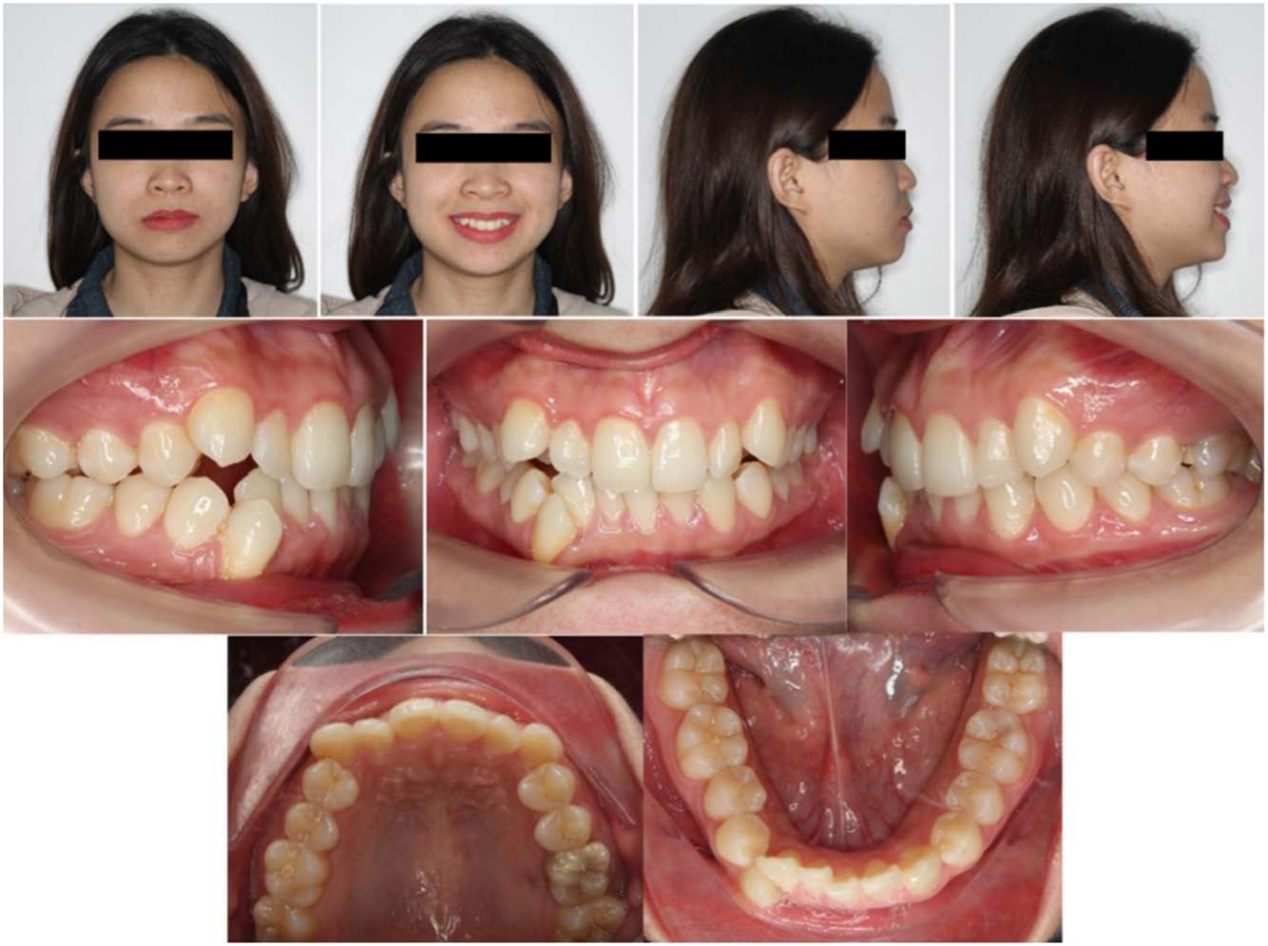
Pretreatment extraoral and intraoral photographs.

The patient is a 26-year-old female who presented with the chief complaint of crowding in the anterior region. Her medical and family history was noncontributory. Extraoral examination revealed facial asymmetry with mandibular deviation to the right and a convex facial profile on the lateral view (Fig. 1). The smile analysis showed 80% upper incisor display on smiling, a low smile line, and narrow buccal corridors.

Intraoral examination revealed a right-side mild Class III molar relationship, with moderate crowding in the maxillary arch and severe crowding in the mandibular arch, including complete space deficiency for the right mandibular canine. The mandibular dental midline was deviated 2 mm to the right. The patient exhibited a 40% deep overbite and an overjet of 1.7 mm. The curve of Spee was deep. The maxillary left first molar presented with pulpal necrosis.

**Figure 2.**
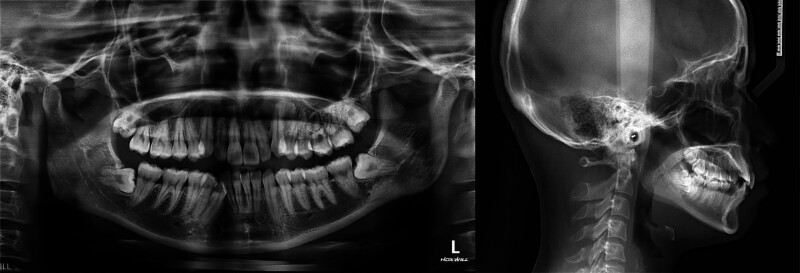
Pretreatment panoramic and cephalometric radiographs.

Cephalometric analysis showed skeletal Class I pattern (Fig. 2). Relevant measurements included an sella-nasion-A point of 81.08°, sella-nasion-B point (SNB) of 77.32°, and A point–nasion–B point of 3.76° (Table 1). The Frankfort mandibular plane angle was 28.03°, indicating a normal mandibular plane angle with no significant vertical skeletal discrepancy. The maxillary incisors were slightly retroclined, while the mandibular incisors were within normal inclination, as shown by upper incisor to sella–nasion (U1-SN) of 97.06° and lower incisor to mandibular plane (L1-MP) of 91.55°.

**Table 1:** Measurements obtained from pretreatment and post-treatment lateral cephalometric radiographs.

Measurements	Pretreatment	Post-treatment	Norm
Skeletal			
SNA (°)	81.08	81.13	81.1 ± 3.7
SNB (°)	77.32	77.41	79.2 ± 3.8
ANB (°)	3.76	3.71	2.5 ± 1.8
FMA (°)	28.03	28.46	25.0 ± 4.0
Wits appraisal (mm)	−1.98	−2.93	0.4 ± 2.3
Dental			
Upper incisor/SN (°)	97.06	100.98	105.3 ± 6.6
Upper incisor/NA (°)	15.98	19.86	22.0 ± 5.0
Upper incisor/NA (mm)	4.15	5.28	4.0 ± 3.0
L1-MP (°)	91.55	98.75	90.0 ± 3.5
Lower incisor/NB (°)	25.18	31.91	25.0 ± 5.0
Lower incisor/NB (mm)	5.18	7.72	4.0 ± 2.0
Interincisal angle (°)	135.08	124.53	128.0 ± 5.3
Overjet (mm)	1.7	1.5	2.0 ± 2.0
Overbite (mm)	3.1	1.5	2.0 ± 2.0
Soft tissue			
Upper lip/E-line (mm)	2.09	1.70	0.0 ± 2.0
Lower lip/E-line (mm)	4.88	4.14	0.0 ± 2.0

ANB =  A point–nasion–B point, FMA = Frankfort mandibular angle, L1-MP = lower incisor to mandibular plane, NA = nasion-point A, NB = nasion-point B, SN = sella-nasion, SNA = sella-nasion-point A, SNB = sella-nasion-point B.

Soft tissue analysis revealed that the upper and lower lips were protrusive relative to the E-line by 2.09 and 4.88 mm, respectively, contributing to a convex facial profile.

Panoramic radiographic evaluation revealed mandibular skeletal asymmetry and mild alveolar bone crest resorption in both arches. All four third molars were impacted. The maxillary left first molar exhibited a periapical and furcal radiolucency.

Based on clinical and radiographic findings, the patient was diagnosed with a unilateral dental Class III subdivision malocclusion, severe crowding in the mandibular arch, moderate crowding in the maxillary arch, a mandibular midline deviation to the right, a poor-prognosis maxillary left first molar with pulpal necrosis, mild lip protrusion with a slightly convex facial profile, and impacted third molars in all four quadrants.

### 2.2. Treatment objectives

The primary treatment objectives were established to comprehensively and systematically address the skeletal, dental, and esthetic concerns identified in this case. From a skeletal perspective, the goal was to maintain the existing skeletal relationship and preserve vertical facial balance. The treatment plan aimed to maintain the current mandibular plane angle and minimize further anterior projection of the maxillomandibular complex.

Dentally, the objectives included alleviating moderate crowding in the maxillary arch and resolving severe crowding in the mandibular arch, particularly the complete blockage of the lower right canine. The maxillary left first molar was indicated for extraction due to non-restorability. Space closure on the left maxilla was planned with reciprocal mechanics – controlled mesialization of the second and third molars together with distalization of the premolars into the extraction space. Simultaneous distalization of the maxillary right and mandibular left posterior segments using TADs was planned to create space and aid in midline correction of both arches. Leveling and alignment of both dental arches were necessary to flatten the deep curve of Spee and achieve ideal intercuspation and arch coordination.

Occlusally, the treatment aimed to correct the unilateral Class III relationship on the right side, achieve midline coordination, and establish optimal overjet and overbite. In the transverse dimension, the goal was to improve posterior arch coordination and increase buccal corridor width, enhancing the esthetics of the smile. From a soft tissue perspective, the objective was to improve facial harmony by retracting the anterior teeth, thereby reducing lip protrusion, alleviating mentalis strain, and enhancing both the smile line and overall profile.

These objectives were designed to achieve long-term stability of the occlusion, enhance facial esthetics, and maintain periodontal health.

### 2.3. Treatment alternatives

Multiple treatment alternatives were carefully evaluated and discussed with the patient, each presenting distinct biomechanical implications, advantages, and limitations.

The first approach involved preserving all existing teeth, including the upper left first molar, which required root canal treatment followed by crown restoration. The goal was to alleviate crowding and achieve alignment without extractions. The primary advantage of this option was the preservation of the full dentition, thus avoiding any tooth removal. However, given the moderate-to-severe crowding – particularly in the mandibular arch – alignment without extractions would result in significant proclination of the anterior teeth. This would exacerbate lip protrusion and yield an overly convex facial profile. Moreover, the long-term prognosis of the maxillary left first molar post-treatment remained questionable. As a result, this option was considered suboptimal in addressing the patient’s chief complaint and improving facial esthetics.

The second treatment alternative involved the extraction of all 4 first premolars to relieve crowding and permit controlled retraction of the anterior teeth. The key benefit of this strategy was its capacity to maintain or even improve soft tissue balance by avoiding further lip protrusion. Nonetheless, in patients with mild skeletal Class III discrepancies, this extraction pattern poses a notable risk of excessive retroclination of the maxillary incisors. Such an outcome could compromise smile esthetics and reduce maxillary incisor display. Additionally, this plan increases the anchorage demands and may prolong the overall treatment duration.

The third option proposed the extraction of three premolars and the maxillary left first molar, which had a hopeless prognosis. This approach addressed the restorative limitations while preserving bilateral symmetry in the mandibular arch. The resultant space distribution allowed for effective alignment and reduction of anterior protrusion. Advantages included the avoidance of endodontic and prosthetic intervention while still permitting acceptable anterior-posterior correction. However, this plan precluded the establishment of a full Class I molar relationship on the maxillary left side and maintained a risk of undesirable retroclination of the maxillary incisors.

The final and selected treatment strategy involved the extraction of maxillary right and mandibular third molars, along with the poor-prognosis maxillary left first molar. Subsequent space closure was planned using conventional anchorage, in combination with miniscrew-assisted distalization of the maxillary right and mandibular left posterior segments. This approach enabled space creation for crowding relief, midline correction, and maxillary retraction without excessively compromising incisor inclination or necessitating prosthetic replacement. The primary advantages of this approach included optimal control of anterior tooth positioning, prevention of incisor proclination or retroclination, and improved soft tissue esthetics. However, the treatment required complex biomechanical management and a high level of patient compliance. Additionally, reliance on TADs introduced potential risks such as screw loosening or limited distalization efficiency.

### 2.4. Treatment progress

Orthodontic treatment was initiated with the placement of lingual metal brackets on the maxillary arch and self-ligating metal brackets on the mandibular arch. Leveling and alignment commenced using a 0.012-inch nickel-titanium (NiTi) archwire, followed by progression to 0.014- and 0.016-inch NiTi archwires. This initial phase aimed to relieve crowding and align both arches.

One month following initial bonding, the maxillary left first molar, the maxillary right third molar, and both mandibular third molars were extracted under local anesthesia. These extractions were planned to manage space, facilitate alignment, and avoid prosthetic replacement. At the 15th month of treatment, 2 TADs were placed: a 1.6 × 10 mm miniscrew in the palatal bone between the maxillary left first and second molars and a 1.8 × 12 mm miniscrew at the external oblique ridge of the mandibular left posterior segment.

**Figure 3.**
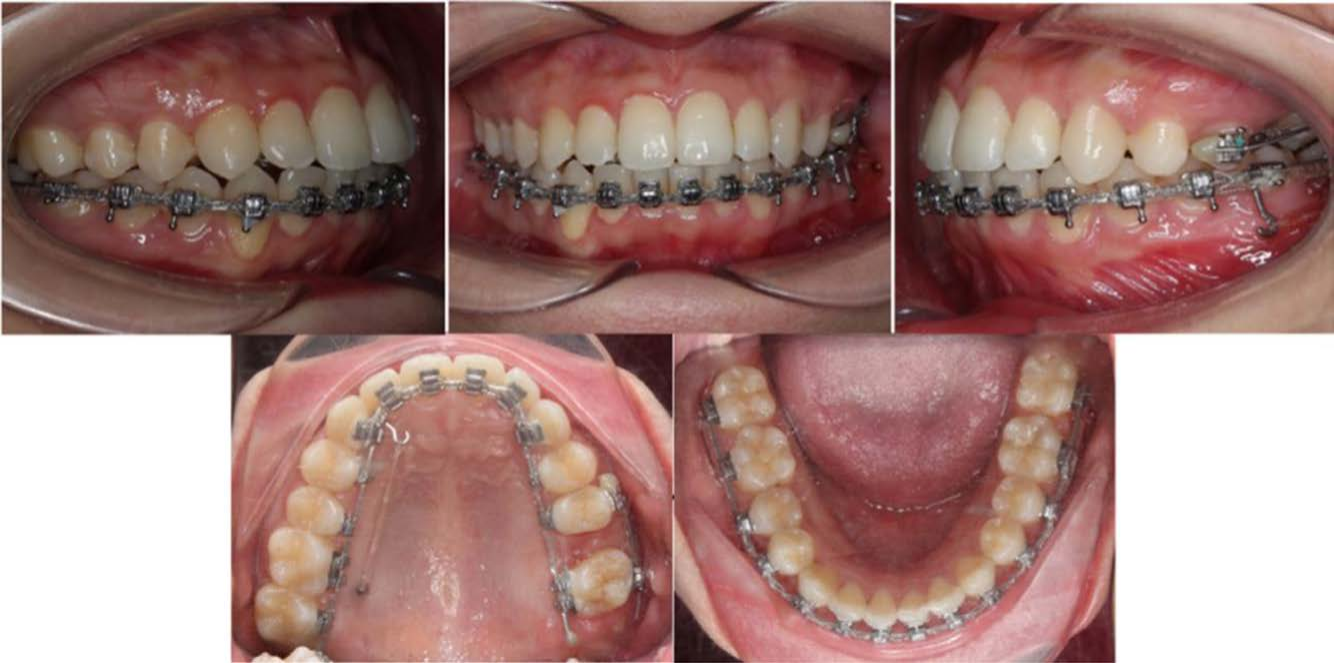
Progress intraoral and extraoral photographs. Space closure progressed by reciprocal mechanics to close the maxillary left first molar extraction space. On the contralateral sides, the maxillary right and mandibular left segments were distalized with miniscrews.

A continuous elastic chain delivered reciprocal forces to mesialize the left second and third molars while simultaneously distalizing the left premolars into the first-molar extraction site (Fig. 3). Concurrently, distalization of the mandibular dentition was initiated using the miniscrew anchorage system on the lower left side. To enhance control of posterior tooth movement, lingual brackets were bonded to the mandibular first and second molars.

After three months of traction of the maxillary left second molar, additional buccal brackets were bonded to the maxillary left second premolar and molar to provide dual-surface force application. A 0.019 × 0.025-inch NiTi archwire was placed, and elastic chains were employed on both buccal and lingual aspects to further protract the maxillary left second molar.

At 12 months, an electrosurgical procedure was performed to expose the partially erupted maxillary left third molar, which was subsequently bonded on the buccal surface. Traction of this tooth was initiated using chain elastics in conjunction with 0.016 × 0.022-inch NiTi archwire and continued with sequential progression to 0.017 × 0.025-inch NiTi and 0.019 × 0.025-inch stainless steel archwires.

To optimize anterior torque control and coordinate the maxillary and mandibular arches, lingual brackets were bonded to the upper anterior teeth. At 15 months, a second palatal miniscrew was placed between the maxillary right first and second molars to reinforce posterior anchorage. En-masse retraction of the anterior segment followed, resulting in improved upper incisor inclination and reduction of lip protrusion.

Throughout treatment, cross-arch elastics were employed to improve inter-arch coordination and molar intercuspation. After 17 months of active treatment, all brackets were removed. Fixed lingual retainers were bonded to both the maxillary and mandibular arches. Residual adhesive was carefully removed using silicone polishing tips to preserve enamel integrity. The patient was transitioned to the retention phase with clear overlay retainers and scheduled for follow-up appointments every 6 months to monitor long-term stability and detect potential relapse.

### 2.5. Treatment results

**Figure 4.**
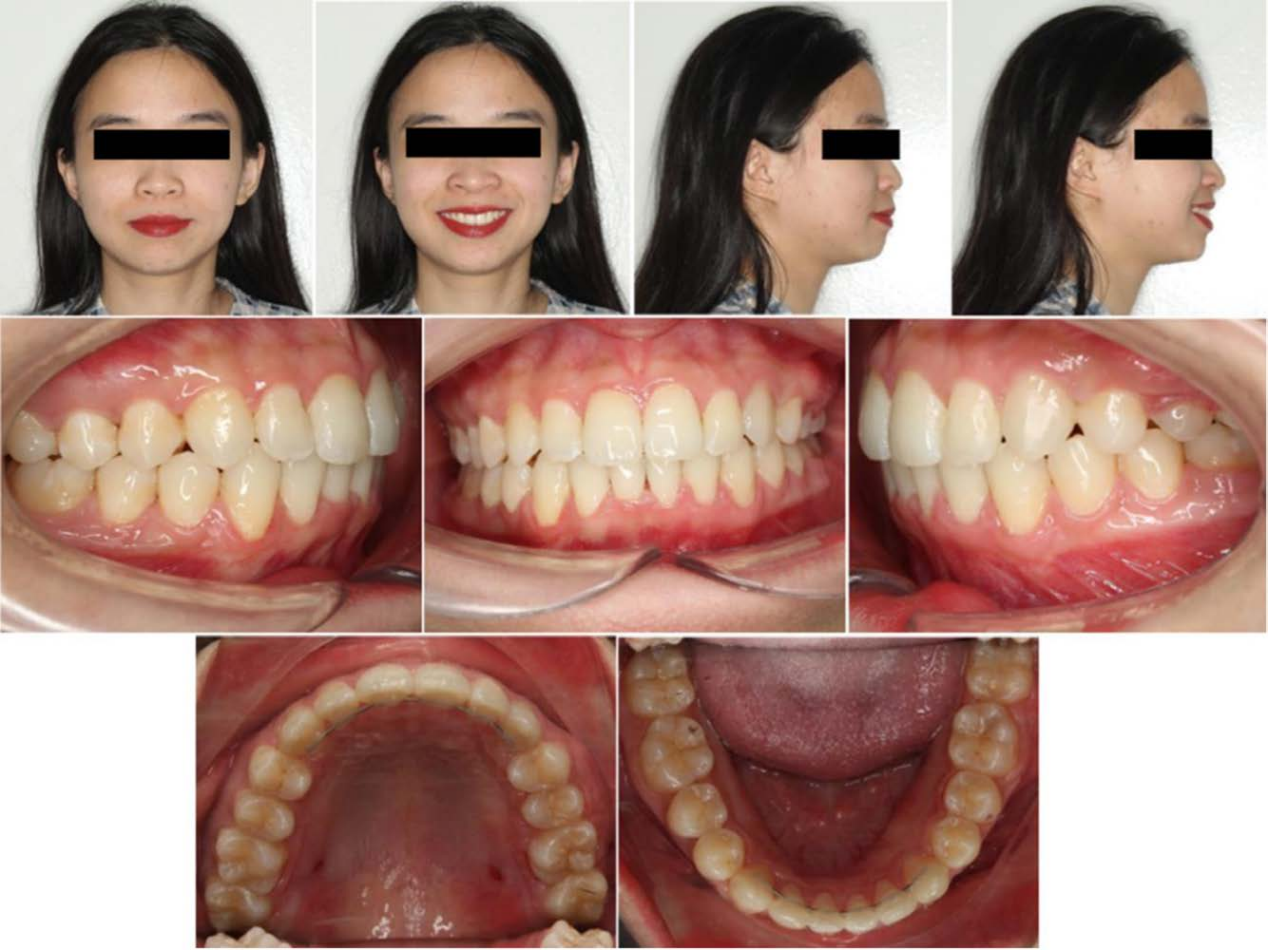
Posttreatment extraoral and intraoral photographs.

The treatment effectively achieved the primary objectives of correcting the Class III subdivision malocclusion, improving dental alignment, and enhancing facial esthetics (Fig. 4).

**Figure 5.**
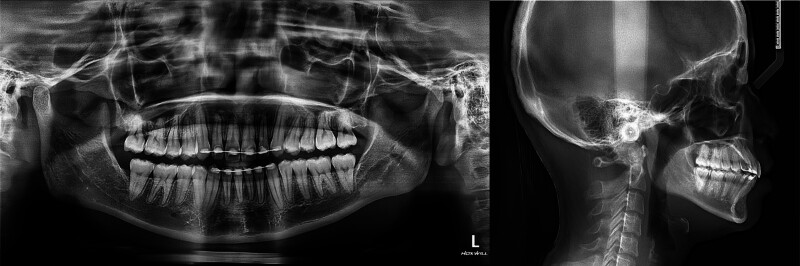
Posttreatment panoramic and cephalometric radiographs.

In the maxillary dentition, significant improvements were made in the alignment. The upper incisors were proclined (U1-SN from 97.06° to 100.98°), which contributed to better aesthetics and correction of the deep bite (Fig. 5). The U1-NA angle and distance increased (19.86° and 5.28 mm, respectively), enhancing the alignment of the maxillary anterior teeth. The upper arch was aligned, with no remaining crowding, and the tooth 26 extraction site was successfully closed. In the mandibular dentition, the lower incisors were proclined (98.75°), and the crowding in the lower arch was completely resolved. The L1-NB angle and distance increased (31.91° and 7.72 mm).

The occlusion was significantly improved, with Class I relationship achieved on the canine and first molar. The midlines of both arches were aligned, and the midline of the face was symmetrical, which contributed to improved facial harmony. The overjet and overbite were normalized, with the anterior bite now in a normal position.

Anchorage loss manifested primarily as controlled incisor proclination (U1-SN + 3.9°; L1-MP + 7.2°), which we accepted to aid deep-bite leveling and space resolution. As planned, reciprocal, dual-surface biomechanics closed roughly half of the space by mesial migration of the left posterior molars and the other half by distal repositioning of the left premolars.

Regarding soft tissue, both the upper and lower lips moved closer to the E-line (1.70 and 4.14 mm), indicating an improvement in lip positioning, enhancing the overall facial esthetics, and providing a more balanced relationship between the lips and the face.

Radiographically, the panoramic x-ray showed no significant changes in the bone levels compared to pretreatment, with the roots of the teeth remaining parallel and no signs of root resorption. This was an important result, ensuring the stability of the treatment.

**Figure 6.**
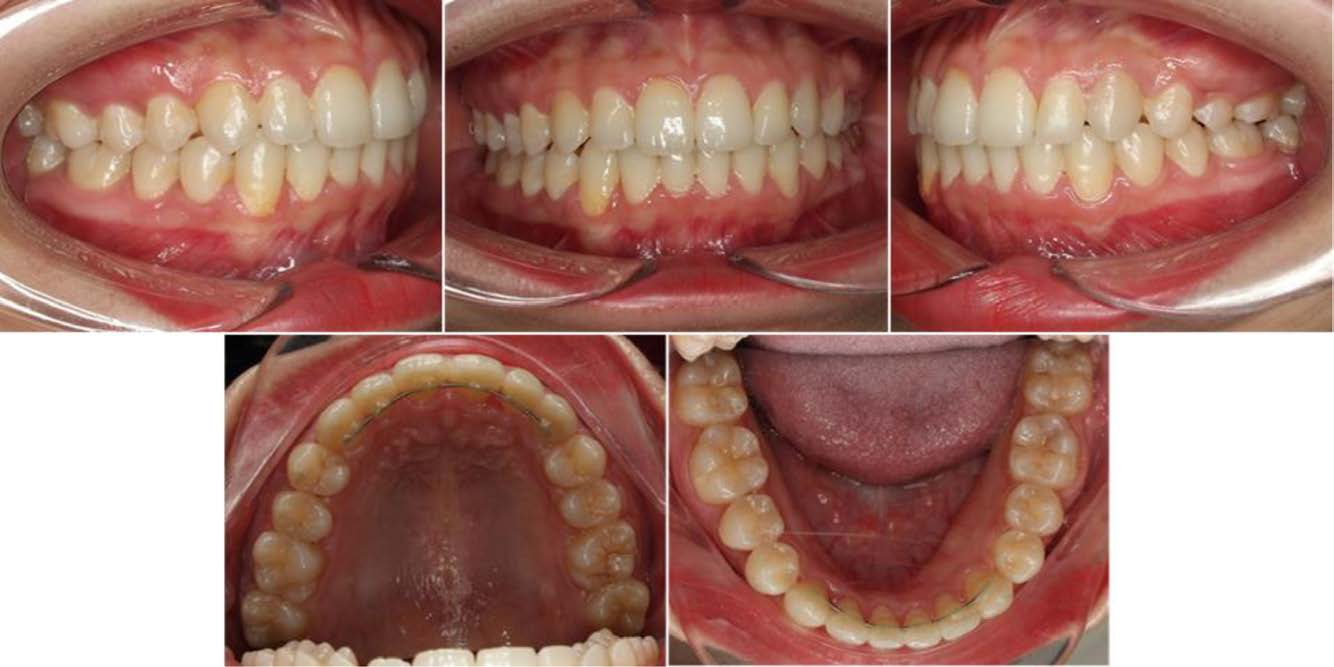
One-year follow-up extraoral and intraoral photographs.

A 1-year retention follow-up demonstrated that the achieved occlusion, midline coordination, overjet, and overbite were maintained (Fig. 6). No clinically relevant relapse of the mandibular midline, posterior intercuspation, or molar space closure was observed at that time.

## 3. Discussion

This case highlights the clinical effectiveness of an asymmetrical extraction approach combined with miniscrew-assisted biomechanics in the management of adult Class III malocclusion characterized by dental midline deviation and unilateral molar loss. The decision to extract the maxillary left first molar and close the resulting space using conventional anchorage, in conjunction with distalization of the maxillary right posterior segment, facilitated overall maxillary retraction. This approach contributed to maintaining maxillomandibular coordination, minimizing the risk of midline deviation, and simultaneously improving occlusal relationships and enhancing facial soft tissue esthetics. In the mandibular arch, unilateral distalization was essential for correcting the lower dental midline, alleviating crowding, and establishing optimal occlusal relationships.

**Figure 7.**
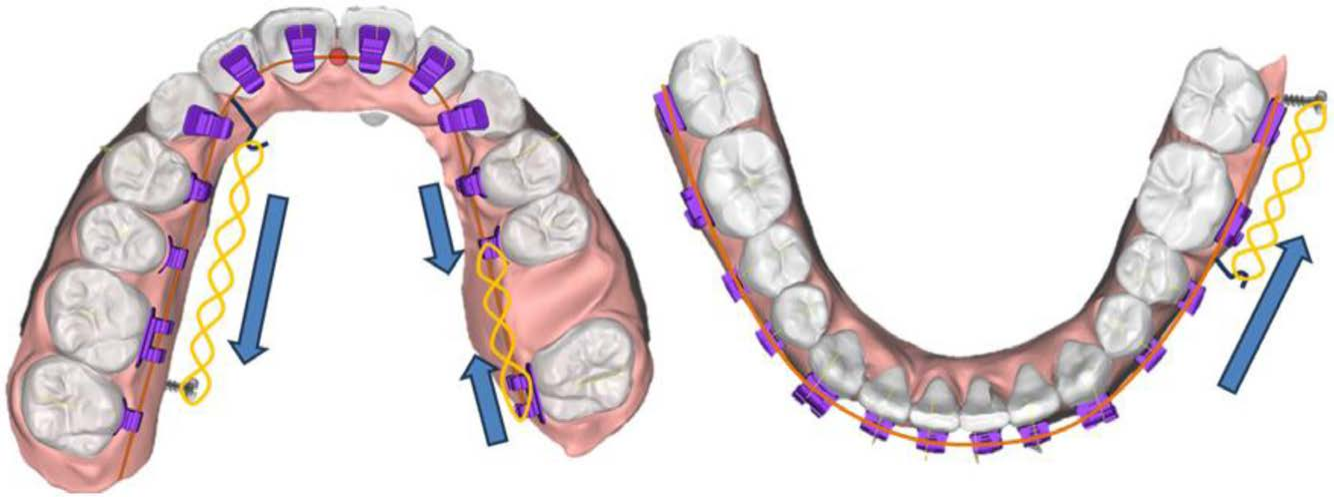
Schematic of unilateral reciprocal mechanics (occlusal views). At the maxillary left extraction site, space closure was produced by reciprocal forces, with arrows denoting mesialization of the molars and distalization of the premolars. On the contralateral sides, miniscrew-assisted distalization of the maxillary right and mandibular left segments coordinated the arches and recentered the midlines. Yellow coils depict the elastic chain.

Unlike prior reports that used either unilateral extraction or one-sided distalization, our case integrates both in opposite quadrants: (i) reciprocal space closure on the maxillary left to substitute the extracted first molar, while (ii) miniscrew-assisted distalization of the maxillary right and mandibular left segments to correct the Class III subdivision and recenter both arches.^[5,6]^ This three-quadrant, TAD-supported asymmetry simultaneously solved severe bimaxillary crowding and a mandibular midline shift without four-premolar extraction, orthognathic surgery, or prosthetic replacement – broadening the clinical playbook for adult Class III camouflage (Fig. 7).

Miniscrews provided reliable skeletal anchorage to support both protraction and distalization forces, enabling controlled tooth movement and minimizing undesired side effects such as tipping or anchorage loss.^[7,8]^ Through meticulous biomechanical control and efficient space management, this treatment approach achieved favorable outcomes in terms of occlusion, esthetics, and function, without resorting to orthognathic surgery or prosthetic replacement of the missing molar. Overall, the combination of asymmetrical extraction and miniscrew-assisted mechanics represents a conservative, non-surgical, and efficient alternative for managing selected adult patients with asymmetrical Class III malocclusion.

To limit unwanted anchorage loss, we used palatal miniscrews to reinforce maxillary posterior anchorage, rectangular SS archwires and torque control anteriorly, and dual-surface force application (buccal and lingual) during left-side mesialization to reduce tipping. Mild incisor proclination was acceptable within an adult camouflage scheme to flatten the curve of Spee and address severe bimaxillary crowding.

In this patient, the skeletal bases were essentially Class I at baseline with a normal vertical pattern and only mild soft-tissue protrusion. The primary treatment objectives were therefore not to induce large skeletal or vertical changes, but rather to eliminate severe bimaxillary crowding, correct the unilateral Class III subdivision and mandibular midline deviation, and improve the facial profile while preserving the existing vertical dimension. Because the biomechanics relied on unilateral maxillary molar extraction and miniscrew-assisted asymmetric distalization of posterior segments, most of the correction was dentoalveolar rather than skeletal. As a result, angular cephalometric changes were modest, which is clinically appropriate in a patient who did not require skeletal correction. The clinically meaningful outcomes were coincident maxillary and mandibular midlines, establishment of a Class I canine relationship, normalization of overjet and overbite, and reduction of lip protrusion.

In adult Class III camouflage, maintaining the basal maxillomandibular relationship and preserving vertical dimension is often advantageous: large skeletal changes are unlikely biologically and may compromise facial balance, while targeted dentoalveolar movements (space redistribution and torque control) predictably normalize overjet, overbite, and midlines with fewer soft-tissue trade-offs.^[9,10]^ Our strategy aligns with adult camouflage series, reporting favorable esthetics and function when asymmetric mechanics are used to correct subdivision patterns without attempting skeletal correction.^[6,11]^

In managing adult patients with asymmetric Class III malocclusion, treatment alternatives must be carefully weighed in terms of esthetic demands, biomechanical feasibility, and long-term stability. Conventional approaches such as four-premolar extraction may risk excessive incisor retroclination and unfavorable profile changes, whereas non-extraction protocols can lead to insufficient space resolution. Asymmetric extraction patterns, particularly involving non-restorable molars, offer a conservative alternative that allows for targeted space management and midline correction. When combined with skeletal anchorage, these strategies enable controlled tooth movement while preserving soft tissue balance and occlusal harmony.

The management of asymmetrical malocclusions often necessitates unconventional mechanics such as unilateral extractions or one-sided distalization. In the literature, there are numerous reports supporting these approaches in non-growing patients. For example, Booij et al^[3]^ demonstrated that a unilateral maxillary first molar extraction can successfully resolve a Class II subdivision discrepancy without the need for headgear, achieving stable occlusion and even facilitating eruption of the third molar on the extraction side. Long-term evaluations of this protocol have confirmed its favorable outcomes and stability by Livas et al^[1]^ In Class III camouflage treatment, selective tooth removal has also been employed to correct asymmetries. Ruellas et al^[12]^ treated an adult Class III subdivision by extracting the mandibular first molars, which provided the space to correct a unilateral anterior crossbite and establish Class I molar relationships. Other authors have reported similar asymmetric extraction strategies, such as removal of a single lower premolar on the affected side to shift the midline and dental occlusion.^[13]^ Even posterior teeth have been targeted, with Jacobs et al^[14]^ describing extraction of lower second molars in a Class III patient with a vertical growth pattern, using the subsequent third-molar eruption to aid in correction. These unconventional extraction protocols indicate that asymmetric tooth removal can produce satisfactory and stable results in carefully selected cases.

The present report differs from previously published asymmetric extraction or unilateral distalization cases in several key respects. First, space creation and space closure occurred in opposite quadrants: the hopeless maxillary left first molar was extracted, and the maxillary left posterior segment was protracted into that space, while the contralateral maxillary right segment and the mandibular left segment were distalized with miniscrew anchorage to correct the Class III subdivision and recenter both arches. This required coordinated asymmetric biomechanics in three quadrants rather than simple unilateral distalization. Second, this protocol simultaneously resolved severe bimaxillary crowding and corrected a mandibular midline deviation without resorting to four-premolar extraction, orthognathic surgery, or prosthetic rehabilitation of the missing molar site. Third, a hybrid appliance strategy was implemented: lingual brackets in the maxillary arch for esthetic, buccal auxiliaries for force application, and skeletal anchorage in both the palate and the mandibular buccal shelf. Collectively, these features illustrate a conservative pathway for adult Class III subdivision patients who present with a single non-restorable molar and significant crowding. This integrated approach – posterior protraction on one side plus contralateral TAD-supported distalization across both arches – has been less emphasized in prior reports and expands the clinical toolbox for managing asymmetric malocclusion in adult patients.

TADs have further expanded the clinician’s ability to correct unilateral discrepancies without compromising anchorage. One notable example is the case by Ma et al,^[6]^ in which a severe unilateral Class III malocclusion with midline deviation was corrected by miniscrew-assisted molar distalization. By distalizing the entire maxillary right and mandibular left segments, they achieved coincident midlines and a balanced occlusion, all without extractions, and maintained stability at a 2-year follow-up. Chen et al^[15]^ likewise reported a nonsurgical Class III treatment that combined strategic extractions with skeletal anchorage: they extracted the lower second molars and placed retromolar mini-implants to distalize the mandibular arch, allowing the third molars to erupt into proper position and obviating the need for premolar extractions. In our patient, we integrated both strategies by performing a unilateral maxillary molar extraction to relieve crowding on one side and en-masse unilateral distalization of the posterior segments (in both upper and lower arches) on the contralateral side with TAD support. This hybrid approach parallels the report of de Lima et al,^[16]^ who combined a one-sided lower premolar extraction with bilateral miniscrews to correct a Class III subdivision in an adult patient. By leveraging asymmetric skeletal anchorage in conjunction with an atypical extraction, the present case achieved correction of the unilateral Class III relationship and midline discrepancy without resorting to orthognathic surgery.

Although 2-dimensional records were sufficient to execute our plan, contemporary digital workflows and 3-dimensional modalities can strengthen planning for asymmetric force systems. Digital pathways help standardize records and improve transfer from planning to delivery, thereby supporting precise application of unilateral mechanics and patient communication.^[17]^ In parallel, simulation methods, such as finite-element analyses on patient-specific geometry, can estimate stress and moment-force patterns for alternative vectors or attachment sites, aiding the selection of reciprocal mechanics while respecting anatomical constraints and minimizing unwanted tipping or rotation. These directions are consistent with recent evidence on digital workflows and dental biomechanics simulation in the broader dental literature.^[18]^

The treatment in this case presents several limitations. First, the decision to perform unilateral molar extraction may not be suitable for all patients with similar Class III malocclusion, especially in cases with more pronounced skeletal discrepancies, as skeletal Class III was not fully corrected and may require surgical intervention for optimal results. Additionally, the use of miniscrew anchorage and distalization techniques requires precise planning and can be challenging if miniscrew failure or patient compliance issues arise. While the biomechanics employed were effective in improving occlusion, the potential for long-term stability remains uncertain, as a longer follow-up period would be needed to assess the durability of the results. The main relapse concerns are midline drift and space reopening at the mesialized maxillary left segment. Furthermore, while the aesthetic outcomes were positive, the proclination of the incisors could pose concerns for some patients, particularly in those with a more convex profile, affecting both esthetic preference and long-term occlusal stability.

## 4. Conclusions

This case report describes the orthodontic management of a 26-year-old female patient who presented with bimaxillary dental crowding, a unilateral Class III malocclusion, and a poor-prognosis maxillary left first molar. Given the dental asymmetry and the compromised prognosis of the affected tooth, unilateral extraction of the maxillary left first molar was indicated. To facilitate space closure and achieve arch coordination, a complex biomechanical approach was employed, involving miniscrew-assisted distalization of the maxillary right and mandibular left posterior segments. This strategy enabled simultaneous space closure, resolution of severe crowding in both arches, correction of midline deviation, and improvement of the occlusal relationship, without requiring prosthetic replacement. The treatment outcome demonstrated a Class I canine relationship, coincident dental midlines, normalized overjet and overbite, and enhanced facial esthetics. This case underscores the effectiveness of asymmetrical mechanics combined with skeletal anchorage in managing unilateral molar extractions and achieving both functional and esthetic objectives in complex malocclusions.

## Author contributions

**Conceptualization:** Viet Anh Nguyen.

**Data curation:** Thi Quynh Phuong Vo.

**Investigation:** Viet Anh Nguyen, Thi Quynh Phuong Vo.

**Methodology:** Viet Anh Nguyen, Thi Quynh Phuong Vo.

**Project administration:** Viet Anh Nguyen.

**Supervision:** Viet Anh Nguyen.

**Validation:** Viet Anh Nguyen.

**Visualization:** Thi Quynh Phuong Vo.

**Writing – original draft:** Thi Quynh Phuong Vo.

**Writing – review & editing:** Viet Anh Nguyen.

## References

[1] Livas C Pandis N Booij JW Katsaros C Ren Y. Long-term evaluation of Class II subdivision treatment with unilateral maxillary first molar extraction. Angle Orthod. 2015;85:757–63. 25386872 10.2319/071614-499.1 PMC8610412

[2] Anh NV Ngoc VTN Son TM. Closure of first molar extraction spaces and correction of Class II malocclusion using anterior bite turbo and Class II elastics: a case report. APOS Trends Orthod. 2025;15:102–7.

[3] Booij JW Livas C. Unilateral maxillary first molar extraction in class II subdivision: an unconventional treatment alternative. Case Rep Dent. 2016;2016:2168367. 27200194 10.1155/2016/2168367 PMC4856937

[4] Nguyen VA Ha TMA. Treatment of a severe class II subdivision malocclusion following failed bimaxillary anterior segment osteotomy: a case report. BMC Oral Health. 2025;25:612. 40259327 10.1186/s12903-025-06000-7 PMC12013146

[5] Livas C Pandis N Booij JW Halazonetis DJ Katsaros C Ren Y. Influence of unilateral maxillary first molar extraction treatment on second and third molar inclination in class II subdivision patients. Angle Orthod. 2016;86:94–100. 25763687 10.2319/100414-710.1 PMC8603973

[6] Ma QL Conley RS Wu T Li H. Asymmetric molar distalization with miniscrews to correct a severe unilateral class III malocclusion. Am J Orthod Dentofacial Orthop. 2016;149:729–39. 27131255 10.1016/j.ajodo.2015.07.042

[7] Nguyen VA Nguyen NA Doan HL Pham TH Doan BN. Management of anterior and posterior crossbites with lingual appliances and miniscrew-assisted rapid palatal expansion: a case report. Medicine (Baltimore). 2024;103:e40832. 39654233 10.1097/MD.0000000000040832 PMC11630943

[8] Hong VTT Lien TTK Tuan PA Viet H. Superior effect of mini-implant anchorage in the treatment of skeletal class II malocclusion. J Orthod Sci. 2024;13:44. 39758114 10.4103/jos.jos_35_24 PMC11698243

[9] Araujo MTS Squeff LR. Orthodontic camouflage as a treatment alternative for skeletal class III. Dental Press J Orthod. 2021;26:e21bbo24. 10.1590/2177-6709.26.4.e21bbo4 PMC8439187 34524381

[10] Sevillano MGC Diaz GJF de Menezes LM Nunes LKF Miguel JAM Quintão CCA. Management of the vertical dimension in the camouflage treatment of an adult skeletal class III malocclusion. Case Rep Dent. 2020;2020:8854588. 32850154 10.1155/2020/8854588 PMC7441420

[11] Janson G de Freitas MR Araki J Franco EJ Barros SE. Class III subdivision malocclusion corrected with asymmetric intermaxillary elastics. Am J Orthod Dentofacial Orthop. 2010;138:221–30. 20691365 10.1016/j.ajodo.2008.08.036

[12] Ruellas AC Baratieri C Roma MB. Angle class III malocclusion treated with mandibular first molar extractions. Am J Orthod Dentofacial Orthop. 2012;142:384–92. 22920705 10.1016/j.ajodo.2011.01.025

[13] D’Anto V Valletta R De Simone V Pisano M Martina S. Clear aligners treatment of class III subdivision with an extraction of a lower bicuspid. Int J Environ Res Public Health. 2023;20:3550. 36834244 10.3390/ijerph20043550 PMC9967822

[14] Jacobs C Jacobs-Muller C Hoffmann V. Dental compensation for moderate Class III with vertical growth pattern by extraction of the lower second molars. J Orofac Orthop. 2012;73:41–8. 22249271 10.1007/s00056-011-0065-9

[15] Chen K Cao Y. Class III malocclusion treated with distalization of the mandibular dentition with miniscrew anchorage: a 2-year follow-up. Am J Orthod Dentofacial Orthop. 2015;148:1043–53. 26672711 10.1016/j.ajodo.2015.03.034

[16] de Lima EM BF Mezomo M Pasquali CE Farret M. Orthodontic treatment of Class III malocclusion with lower extraction and anchorage with mini implants: case report. J World Federation Orthodontists. 2017;6:28–34.

[17] Senthilvel Palani B Rathee M Tomar SS Singla S. Clinical outcomes of traditional versus digital prosthetic workflows following immediate loading of implants in esthetic zone: a systematic review and meta-analysis. J Prosthet Dent. 2025;134:S0022–3913. 10.1016/j.prosdent.2025.09.005 41076437

[18] Divakar S Rathee M Jain P Malik S Tomar SS Alam M. Comparative evaluation of mechanical effects of two designs of immediately placed customized root-analogue zirconia implants in the maxillary and mandibular posterior regions: a finite element analysis. Dent Med Probl. 2025;62:99–106. 39998333 10.17219/dmp/152315

